# Evidence into practice: a national cohort study of NICE-recommended oncological drug therapy utilisation among women diagnosed with invasive breast cancer in England

**DOI:** 10.1038/s41416-023-02439-z

**Published:** 2023-09-23

**Authors:** Melissa Ruth Gannon, David Dodwell, Ajay Aggarwal, Min Hae Park, Katie Miller, Kieran Horgan, Karen Clements, Jibby Medina, David Alan Cromwell

**Affiliations:** 1 Department of Health Services Research & Policy, London School of Hygiene & Tropical Medicine, London, UK; 2 Clinical Effectiveness Unit, The Royal College of Surgeons of England, London, UK; 3 Nuffield Department of Population Health, University of Oxford, Oxford, UK; 4 Department of Oncology, Guys Cancer Centre, Guy’s & St Thomas’ NHS Trust, London, UK; 5 Department of Breast Surgery, St James’s University Hospital, Leeds, UK; 6 NHS England, 5th Floor, 23 Stephenson Street, Birmingham, UK

**Keywords:** Breast cancer, Epidemiology, Epidemiology, Ageing

## Abstract

**Background:**

Multiple drug treatments are approved for invasive breast cancer (IBC). We investigated uptake of NICE-recommended oncological drugs and variation by age, comorbidity burden and geographical region.

**Methods:**

Women (aged 50+ years) diagnosed with IBC from 2014 to 2019, were identified from England Cancer Registry data and drug utilisation from Systemic Anti-Cancer Therapy data. Interrupted time series analysis assessed national-level changes in drug use after publication of NICE recommendations. Regression models analysed variation in use.

**Results:**

This national cohort included 168,449 women. Use of drugs recommended for first-line treatment varied, from 26.6% for CDK 4/6 inhibitors to 63.8% for HER2-targeting therapies. Utilisation of drugs with a NICE recommendation published between 2014 and 2019, increased among patients diagnosed around the time of publication, except in the case of pertuzumab for metastatic breast cancer (MBC) which was previously accessible via the Cancer Drugs Fund (though use of pertuzumab for MBC increased from 34.1% to 75.0% across the study period). Use of trastuzumab and neoadjuvant/adjuvant pertuzumab varied by geographical region. Use was low for ribociclib (2.2%), abemaciclib (2.3%) and for drugs recommended beyond the first-line setting. For all drugs, use after NICE recommendation varied by age at diagnosis and increased as stage increased.

**Conclusions:**

Use of NICE-recommended drugs for IBC in routine care is variable, with lowest use among women aged 70+ years. Improving access to effective treatments is an important step in improving outcomes.

## Background

The National Institute for Health and Care Excellence (NICE), established in 1999, is responsible for providing evidence-based guidance to support commissioning decisions within the National Health Service (NHS) in England [1]. NICE conducts health technology assessments as part of a technology appraisal (TA) process. The TA process assesses the clinical and cost-effectiveness of drugs submitted for approval and determines which drugs should be funded for use in the routine care of patients, including those with cancer [2, 3]. Based on the findings of this TA process, NICE publishes guidance on the recommended use of the appraised treatment. Drugs recommended by NICE are routinely commissioned by the NHS and should form part of the treatment options for patients.

Over the past two decades, multiple treatments for invasive breast cancer (IBC) have been recommended by NICE. However, there is limited information on the translation of these recommendations into use of the drugs among eligible patients, particularly at a national level [4–9]. Improving the outcomes of cancer treatment requires the translation of national recommendations on optimal treatment into delivery of those drugs to patients. Understanding the utilisation of such treatments in the patient population they were intended for is a vital first step in understanding this process.

This study aimed to investigate the utilisation of NICE-recommended oncological drugs for IBC in routine care, among women aged 50 years and over diagnosed with IBC in England from 2014 to 2019. We also describe the extent to which utilisation varied by age at diagnosis, comorbidity burden and geographical region.

## Methods

### Data sources and study population

This population-based, national cohort study was undertaken as part of the National Audit of Breast Cancer in Older Patients (NABCOP).

#### Identification of NICE-recommended drugs

NICE Technology Appraisal Guidance (TAGs) published from January 2002 (the first TAG published for IBC) to 31 December 2019 were reviewed on 17 March 2022. We identified drugs recommended by NICE for use in routine care. Full details of the process of identifying NICE-recommended drugs can be found in Appendix 1.

#### Routine data

Data on all aspects of diagnosis and care for patients with cancer are routinely collected by the NHS as part of their care and support. This study used pseudonymised cancer registration patient records for all women aged 50 years and over diagnosed with breast cancer in England between January 2014 and December 2019. Data were linked at tumour-level to the Cancer Outcomes and Services Dataset (COSD), which provided information on patient and tumour characteristics, and Hospital Episode Statistics Admitted Patient Care (HES-APC) data for surgical and comorbidity details. Records were linked at patient-level to data in the Primary Care Prescription Database, for information on endocrine therapy use [10]. Linkage of tumour-level records to Systemic Anti-Cancer Therapy (SACT) data provided information on prescribed oncological drugs [11]. SACT data were available for drugs with an administration date from 1 January 2014 up to 28 February 2022. We used SACT data to flag use of oncological drugs recommended by NICE, based on a record of either the drug’s brand or generic name in the drug name field. For analysis involving trastuzumab, Herceptin and other trastuzumab biosimilars (herzuma; ontruzant; trazimera) were included [12]; trastuzumab-emtansine was considered separately because it had its own NICE TAG.

#### Patient inclusion/exclusion

Women were included in the study if they had a registered diagnosis of IBC (ICD-10 code C50) and recorded stage 1–4. Women were excluded if they had a date of death within six months of diagnosis or a previous registration of breast cancer.

### Definition of study variables

Data on the following patient demographics and tumour characteristics were taken from Cancer Registry and COSD: age at diagnosis (years), ethnicity (White, Mixed, Asian or Asian British, Black or Black British, Other Ethnic Group, Not Reported), level of deprivation, overall stage (1, 2, 3a, 3b, 3c, 4), tumour stage (T1, T2, T3, T4), nodal stage (N0, N1–3), invasive grade (G1, G2, G3), estrogen receptor (ER)/progesterone receptor (PR)/human epidermal growth receptor 2 (HER2) status (positive or negative). Deprivation was measured using the Index of Multiple Deprivation 2019 rank, derived from the patient’s postcode at diagnosis, with ranks assigned to national quintiles of deprivation, from most (group 1) to least (group 5) deprived. This allocation was done by NCRAS and the calculated deprivation quintile provided within Cancer Registry data.

Stage groups were defined as early invasive breast cancer (EIBC; stage 1–3a), locally advanced breast cancer (LABC; stage 3b–c) and metastatic breast cancer (MBC; stage 4).

IBC was defined as hormone receptor-positive if either ER or PR status were recorded as positive.

Comorbidity burden (0, 1, 2+) was calculated according to the Royal College of Surgeons of England Charlson Comorbidity Index [13]. This counts the presence of specific chronic medical conditions (excluding malignancy) which are identified using ICD-10 diagnosis codes recorded in HES-APC data for episodes in the two years prior to diagnosis.

To identify the use of drugs recommended by NICE as part of first-line treatment, we counted any administration of the drug recorded in SACT either within 12 months of diagnosis or within the first treatment episode (defined by consecutive treatments with no more than an 8 month break between them). For drugs recommended by NICE for use in a surgical setting, use was defined as (i) neoadjuvant where the earliest recorded administration date was within six months of diagnosis and prior to surgery or (ii) adjuvant where the earliest recorded administration date was within six months after the date of surgery. For drugs recommended by NICE for relapse/recurrence, progression or after previous treatment, any drug administration after diagnosis was counted.

### Statistical analysis

All data preparation and statistical analyses were conducted using Stata version 17.0.

Descriptive statistics were used to summarise the percentage (number) of eligible women initiating each NICE-recommended drug. Eligibility for each drug was defined based on stage group and HER2 and/or hormone receptor status as applicable. Use of each NICE-recommended drug was described overall and within patient subgroups defined by stage group, age, year of diagnosis and time from diagnosis to first record.

Multilevel mixed-effects (MLME) logistic regression models were used to analyse differences in drug utilisation across patient subgroups defined by age, comorbidity burden and geographical region, for drugs recommended as part of first-line treatment. MLME models were fitted among eligible women diagnosed after publication of the NICE TAG (referred to as “post-publication” in the results). Models were adjusted for factors associated with treatment decision-making including stage group (as applicable), tumour stage, nodal stage, HER2/hormone receptor status (as applicable) and grade. Deprivation and ethnicity were also included in the models. Missing values were included as unknown categories. MLME models accounted for clustering of patients within Government Office Regions (GORs). Each GOR was fitted as a random intercept, representing differences between GORs not explained by the factors in the model. NHS trusts were aggregated into GOR, due to relatively low levels of activity at NHS trust level, to understand variation by geographical region. MLME models were only fitted where rates of use among eligible women diagnosed after NICE TAG publication were at least 5%, to allow for robust estimates.

For drugs with a NICE TAG published during the period when patients in the study were diagnosed (January 2014 to December 2019), we used interrupted time series analysis (ITSA) to examine the impact of NICE guidance on national-level uptake. ITSA was only used for drugs recommended for use as part of first-line treatment and where rates of use among eligible women diagnosed after NICE TAG publication were at least 5%. The ITSA model allowed for rate of drug initiation to change smoothly over time, as well as abruptly following NICE TAG publication, and for changes due to seasonality [9, 14]. The ITSA model for each drug defined an “intervention” time point that began with the first full month following NICE TAG publication. The model also incorporated a short “transition” period (defined as 6 months prior to NICE TAG publication for neoadjuvant drugs and 12 months otherwise) to account for the fact that the time-series was defined based on month of diagnosis and treatment could occur months later. We used the ‘itsa’ command in Stata to test for statistical evidence of changes in the monthly trends in the number of patients starting treatment from one time period to the next (i.e., pre-publication, transition, post-publication) [15]. Seasonality was adjusted for by including the calendar month as an independent variable in the models. Monthly rates of drug initiations were calculated as the number of eligible women with a record of the drug starting in SACT divided by the number of eligible women diagnosed in the month, multiplied by 1000 (to give rates per 1000 women).

## Results

### Patient cohort

Among 209,968 women aged 50 years and over diagnosed with breast cancer in England from 1 January 2014 to 31 December 2019, 80.2% (*N* = 168,449) had invasive disease with a recorded stage, and did not die within six months of diagnosis (Fig. A1). Of this cohort, women were predominantly diagnosed with EIBC (91.8%), whilst 4.3% had LABC and 3.9% had MBC. Nearly two-thirds were aged 50–69 years at diagnosis (61.3%), whilst 22.9% were 70–79 years and 15.9% were 80+ years.

We identified 13 TAGs which recommended oncological drugs for use in IBC (Table 1). Of these, one TAG had been updated and replaced by NICE guideline NG101 [16, 17]. An additional six published TAGs of oncological drug regimens including bevacizumab, fulvestrant, and lapatinib, were identified where NICE did not recommend treatment and so were excluded from this study (Appendix 1) [18–23].

**Table 1:** Overview of NICE technology appraisal guidance with a positive recommendation published for invasive breast cancer.

Drug (Brand name, Company)	Type	Summary of the indication/eligibility	NICE TAG Publication date (TA#)	Licensing dates [4]		Notes
				FDA	EMA	
Trastuzumab (Herceptin, Roche)	HER2-targeting therapy	HER2-positive metastatic breast cancer	March 2002 (TA34) [43]	1998	2000	-
		HER2-positive early breast cancer	August 2006 (TA107) [16]			Replaced by NG101 [17]
Gemcitabine (Gemzar, Eli Lilly and Company Ltd)	Chemotherapy drug	Metastatic breast cancer which has relapsed following adjuvant/neoadjuvant chemotherapy (including an anthracycline). In combination with paclitaxel.	January 2007 (TA116) [44]	2004	Pre-EMA	-
Pertuzumab (Perjeta, Roche)	HER2-targeting therapy	HER2-positive locally advanced, inflammatory or early breast cancer at high risk of recurrence. Neoadjuvant use, in combination with trastuzumab & docetaxel.	December 2016 (TA424) [45]	2012	2013	Provided with the discount agreed in the PAS.
		HER2-positive metastatic or locally recurrent unresectable breast cancer, not previously treated with anti-HER2 therapy or chemotherapy for metastatic disease. In combination with trastuzumab & docetaxel.	March 2018 (TA509) [46]			Previously on the CDF [47]. Provided within the agreed commercial access arrangement
		HER2-positive (locally advanced, inflammatory or) early breast cancer at high risk of recurrence (lymph node positive). In combination with trastuzumab & chemotherapy.	March 2019 (TA569) [48]			Provided according to the commercial agreement.
Everolimus (Afinitor, Novartis Pharmaceuticals)	mTOR kinase inhibitor	HER2-negative, hormone receptor-positive advanced breast cancer that has recurred or progressed after a non-steroidal aromatase inhibitor. In combination with exemestane	December 2016 (TA421) [49]	2012	2012	Provided with the discount agreed in the PAS.
Eribulin (Halaven, Eisai and Merck)	Chemotherapy drug	Locally advanced or metastatic breast cancer which has had 2 or more rounds of chemotherapy.	December 2016 (TA423) [50]	2010	2011	Provided with the discount agreed in the PAS.
Trastuzumab-emtansine (Kadcyla; Roche)	HER2-targeting therapy	HER2-positive, unresectable locally advanced or metastatic breast cancer previously treated with trastuzumab and a taxane, separately or in combination.	July 2017 (TA458) [51]	2013	2013	Provided with the discount agreed in the PAS.
Palbociclib (Ibrance, Pfizer)	CDK inhibitor	Hormone receptor-positive, HER2-negative locally advanced or metastatic breast cancer as initial endocrine-based therapy with an aromatase inhibitor.	December 2017 (TA495) [52]	2015	2016	Provided with the discount agreed in the PAS.
Ribociclib (Kisqali, Novartis)	CDK inhibitor	Hormone receptor-positive, HER2-negative locally advanced or metastatic breast cancer as initial endocrine-based therapy with an aromatase inhibitor.	December 2017 (TA496) [53]	2017	2017	Provided with the discount agreed in the PAS.
Abemaciclib (Verzenios, Eli Lilly),	CDK inhibitor	Hormone receptor-positive, HER2-negative locally advanced or metastatic breast cancer as initial endocrine-based therapy with an aromatase inhibitor.	February 2019 (TA563) [54]	2017	2018	Provided according to the commercial agreement.
Neratinib (Nerlynx, Pierre Fabre)	HER2-targeting therapy	Extending adjuvant treatment of hormone receptor-positive, HER2-positive early breast cancer where trastuzumab was completed within the previous year.	November 2019 (TA612) [55]	2017	2018	Provided according to the commercial agreement.

*TAG* technology appraisal guidance, *FDA* food and drug administration, *EMA* european medicines agency, *HER2* human epidermal growth receptor 2, *PAS* patient access scheme, *CDF* cancer drugs fund, *mTOR* mechanistic target of rapamycin, *CDK* cyclin dependent kinase, *MAA* managed access agreement.

### Utilisation of drugs recommended by NICE for first-line treatment

Table 2A describes the percentage of eligible women with a record of starting each of the drugs recommended by NICE for use as part of first-line treatment. Rates of use for HER2-targeting therapies were found to be the highest, regardless of age, with 63.8% of women (all stages) with HER2-positive IBC having a record of receiving trastuzumab or pertuzumab as part of first-line treatment (63.3–64.1% EIBC/LABC; 71.8% MBC). Among women newly-diagnosed with HER2-negative, hormone receptor-positive LABC or MBC from 2018 to 2019, 26.6% received one of the three recommended CDK4/6 inhibitors (7.8% LABC; 49.4% MBC). Palbociclib was most frequently used, accounting for 87.2% of CDK4/6 inhibitor-based first-line treatment.

**Table 2:** Frequency of drug use recorded within SACT, for oncological drugs for breast cancer recommended by NICE

A. Oncological drugs for breast cancer recommended by NICE for use as first-line treatment following initial diagnosis															
Drug (NICE TAG date)	Indication/eligibility		Use among women diagnosed 2014–2019						Use among women diagnosed post-publication						Note
			Overall use % (n/N)	% by year of diagnosis *(column %)*					% overall and by age at diagnosis *(column %)*					% starting < 12 m of diagnosis *(row %)*	
				2014/15	2016	2017	2018	2019	Overall	50–59 years	60–69 years	70–79 years	80+ years		
Trastuzumab (Mar-2002)	HER2-positive MBC		71.8% (*n* = 669/932)	60.1%	70.7%	77.3%	80.8%	82.3%	71.8%	80.4%	79.8%	67.2%	44.3%	98.8%	
Trastuzumab (Aug-2006)	HER2-positive	EIBC	63.2% (*n* = 9355/14808)	55.7%	63.5%	67.5%	67.5%	69.9%	63.2%	78.0%	70.7%	52.3%	9.1%	98.9%	
		LABC	64.1% (*n* = 742/1157)	62.6%	65.9%	63.8%	62.7%	68.1%	64.1%	83.8%	77.7%	63.3%	23.5%	98.0%	
Pertuzumab (Dec-2016)	HER2-positive	EIBC	15.0% (*n* = 2049/13686)	0.4%	5.8%	25.8%	28.7%	30.9%	28.4%	38.4%	30.1%	16.8%	1.6%	100.0%	Neoadjuvant use, in combination with trastuzumab & docetaxel. (99.8% recorded as starting chemo & trastuzumab).
		LABC	22.6% (*n* = 214/947)	4.1%	13.4%	41.4%	45.7%	38.8%	42.1%	60.3%	59.6%	28.2%	6.4%	100.0%	
Pertuzumab (Mar-2018)	HER2-positive MBC		52.0% (*n* = 485/932)	34.1%	49.7%	60.2%	61.7%	75.0%	68.4%	87.0%	77.4%	56.5%	24.2%	99.4%	Use for breast cancer not previously treated with anti-HER2 therapy or chemo, in combination with trastuzumab & docetaxel.
Pertuzumab (Mar-2019)	HER2-positive	EIBC	2.1% (*n* = 221/10456)	0.9%	0.6%	0.6%	1.4%	10.3%	11.4%	14.9%	10.7%	11.0%	1.8%	100.0%	Adjuvant use, in combination with trastuzumab & chemo. (93.9% had node-positive tumours).
		LABC	7.0% (*n* = 39/560)	3.8%	2.6%	3.9%	7.6%	28.4%	28.3%	25.0%	80.0%	15.4%	6.7%	100.0%	
Palbociclib (Dec-2017)	HER2-negative, hormone receptor-positive	LABC	2.9% (*n* = 94/3261)	0.0%	0.9%	4.3%	4.0%	8.8%	6.2%	7.0%	4.8%	9.2%	3.5%	89.6%	Use as initial endocrine-based therapy with an AI.
		MBC	17.5%; (*n* = 497/2843)	0.3%	2.2%	18.9%	42.6%	45.0%	43.8%	50.7%	56.9%	48.0%	15.7%	98.2%	
Ribociclib (Dec-2017)	HER2-negative, hormone receptor-positive	LABC	0.3% (*n* = 10/3261)	0.0%	0.2%	0.2%	1.2%	0.2%	0.7%	1.8%	0.0%	1.0%	0.0%	50.0%	Use as initial endocrine-based therapy with an AI.
		MBC	1.7% (*n* = 48/2843)	0.1%	0.0%	2.2%	3.1%	5.0%	4.1%	5.8%	5.2%	2.5%	3.1%	97.2%	
Abemaciclib (Feb-2019)	HER2-negative, hormone receptor-positive	LABC	0.6% (*n* = 19/3261)	0.0%	0.4%	0.6%	1.4%	1.2%	0.9%	0.0%	2.1%	0.0%	2.0%	75.0%	Use as initial endocrine-based therapy with an AI.
		MBC	1.2% (*n* = 35/2843)	0.0%	1.2%	1.2%	1.6%	3.6%	3.8%	3.7%	3.4%	5.0%	2.6%	100.0%	

B. Oncological drugs for breast cancer recommended by NICE for use following relapse/recurrence, progression or previous treatment															
Drug (NICE TAG date)	Indication/eligibility		Use among women diagnosed 2014–2019						Use among women diagnosed post-publication						Note
			Overall use % (*n*/*N*)	% by year of first cycle, among women starting treatment *(row %)*					% by age at diagnosis *(column %)*					% starting 12 + m of diagnosis (row %)	
				2014/15	2016	2017	2018	2019 +	Overall	50–59 years	60–69 years	70–79 years	80+ years		
Gemcitabine (Jan-2007)	MBC		1.2% (*n* = 70/5739)	12.9%	15.7%	17.1%	21.4%	32.9%	1.2%	2.2%	1.5%	1.0%	0.0%	51.4%	Use for relapse following adjuvant/neoadjuvant chemo.
Everolimus (Dec-2016)	HER2-negative, hormone receptor-positive MBC		5.2% (*n* = 134/2576)	4.5%	12.7%	17.9%	20.9%	44.0%	4.4%	6.6%	6.1%	3.4%	1.2%	85.5%	Use for recurrence or progression after a non-steroidal AI.
Eribulin (Dec-2016)	LABC		1.3% (*n* = 89/6632)	5.6%	12.4%	11.2%	15.7%	55.1%	1.0%	2.2%	1.1%	0.8%	0.0%	97.0%	Use for breast cancer which has had 2 or more rounds of chemo.
	MBC		3.3% (*n* = 189/5739)	3.7%	7.4%	12.2%	19.6%	57.1%	3.1%	6.0%	3.1%	2.9%	0.0%	95.5%	
Trastuzumab-emtansine (Jul-2017)	HER2-positive	LABC	8.4% (*n* = 92/1089)	5.4%	10.9%	20.7%	21.7%	41.3%	7.0%	10.8%	10.6%	3.5%	2.4%	76.0%	Use for unresectable breast cancer previously treated with trastuzumab + a taxane
		MBC	22.6% (*n* = 191/846)	6.8%	10.5%	14.7%	20.4%	47.6%	18.9%	23.7%	16.3%	18.6%	10.5%	77.4%	
Neratinib (Nov-2019)	HER2-positive, hormone receptor-positive EIBC		1.1% (*n* = 111/9960)	0.0%	0.0%	0.0%	1.8%	98.2%	3.5%	5.4%	3.2%	0.0%	0.0%	100.0%	Use where trastuzumab was completed within the previous year

*TAG* technology appraisal guidance, *HER2* human epidermal growth receptor 2, *MBC* metastatic breast cancer, *EIBC* early invasive breast cancer, *LABC* locally advanced breast cancer, *chemo* chemotherapy, *AI* aromatase inhibitor.

For all drugs, post-publication use varied. Use was highest among women aged 50–69 years (Table 2A) and increased with increasing stage group (if use was recommended across stage groups). The findings for each drug are presented in the following sections.

#### Trastuzumab

Trastuzumab, a HER2-targeting therapy, was recommended by NICE for HER2-positive MBC in 2002, and HER2-positive EIBC or LABC in 2006 (included in the 2009 NICE guideline); both recommendations were published prior to 2014, the first year for which the study had data, and so ITSA was not possible. Among eligible women 63.7% (*n* = 10,766/16,897) had a record of trastuzumab, with higher use for MBC (71.8%). Use increased over time, most prominently for EIBC and MBC (Fig. A2a). There was an increase in recorded use of trastuzumab biosimilars among women diagnosed from July 2018 onwards (Fig. A2b), forming 18.3% of trastuzumab-based treatment among eligible women diagnosed in 2019 (15.9% EIBC; 33.3% LABC; 39.2% MBC). Age and comorbidity burden were independently associated with use, and there was evidence of variation across GORs (observed range: 58.4–69.6%) (Table A1and A2), from a MLME model. Use decreased with increasing age across all GORs (Fig. A2c). Use had not increased over time among women aged 80+ years (Fig. A2d).

#### Pertuzumab

Pertuzumab is a HER2-targeting therapy recommended by NICE for neoadjuvant use for HER2-positive EIBC or LABC in 2016, for HER2-positive MBC in 2018, and for adjuvant use for HER2-positive EIBC or LABC in 2019; all recommendations were published during the study period and ITSA was possible. The following sub-sections describe use associated with each NICE TAG recommendation.

##### Neoadjuvant use for newly-diagnosed HER2-positive EIBC or LABC

**Fig. 1:**
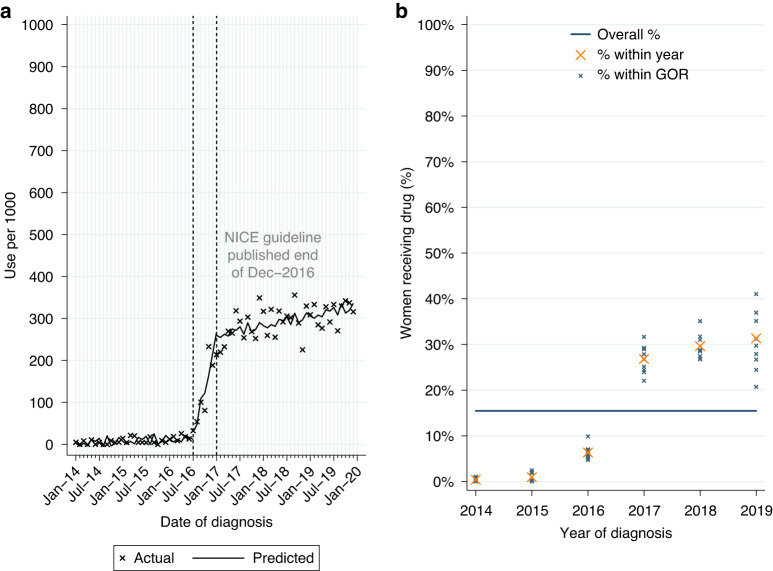
Neoadjuvant pertuzumab utilisation. Neoadjuvant pertuzumab utilisation among women diagnosed with HER2-positive early invasive or locally advanced breast cancer from 2014 to 2019, and receiving surgery within 12 m of diagnosis. **a** Interrupted time series analysis of monthly neoadjuvant pertuzumab initiations. **b** Observed percentage of women initiating neoadjuvant pertuzumab, by Government Office Region (GOR) and year of diagnosis.

Among women who had surgery for HER2-positive EIBC or LABC within 12 months of diagnosis, 15.5% (*n* = 2263/14,633) had a record of neoadjuvant pertuzumab. Use was higher for LABC (22.6%; Table 2A). From ITSA we found an increase in monthly initiations of neoadjuvant pertuzumab of 37.2 patients per 1000 women diagnosed from July to December 2016, with continued increase in monthly initiations of 1.9 patients per 1,000 among women diagnosed post-publication (Table A3, *p* < 0.0001; Fig. 1a). Use increased over time across all GORs (Fig. 1b).

Among eligible women diagnosed post-publication 29.2% had a record of neoadjuvant pertuzumab, with higher use for LABC (42.1%; Table 2A). Fitting a MLME model, age, comorbidity burden and GOR (observed range: 24.6–35.1%; Table A2) were all found to be independently associated with variation in use (Table A1). Use decreased as age increased across all GORs (Fig. A3a).

##### Use for newly-diagnosed HER2-positive MBC

Among 932 women with HER2-positive MBC at diagnosis, 52.0% had a record of pertuzumab (Table 2A). Use increased over time from 34.1% among women diagnosed in 2014–5 to 75.0% in 2019. Of women starting pertuzumab 93.0% also had a record of treatment with trastuzumab and docetaxel, in line with NICE guidance.

**Fig. 2:**
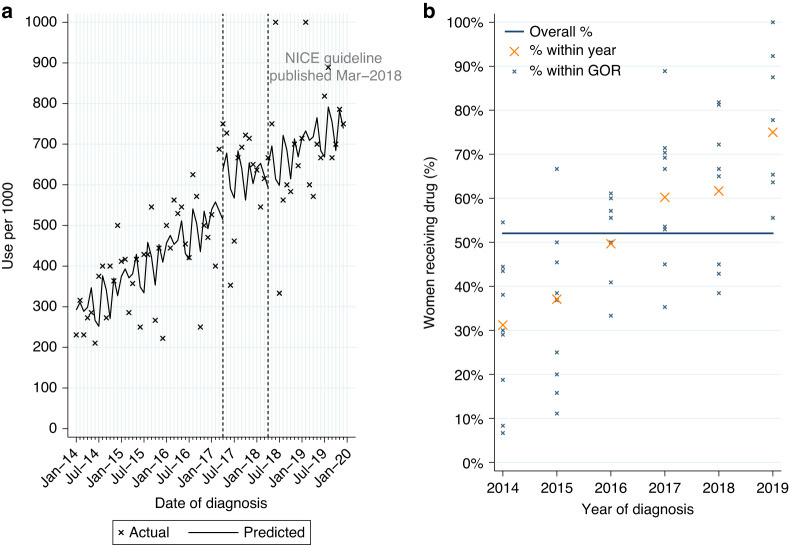
Pertuzumab utilisation for metastatic breast cancer. Pertuzumab utilisation among women initially diagnosed with HER2-positive metastatic breast cancer from 2014 to 2019. **a** Interrupted time series analysis of monthly pertuzumab initiations. **b** Observed percentage of women initiating pertuzumab, by Government Office Region (GOR) and year of diagnosis.

From ITSA, there was no evidence of a monthly increase in initiations among women diagnosed post-publication (Table A3, *p* = 0.33; Fig. 2a). Use increased over time across all GORs (Fig. 2b).

Among eligible women diagnosed post-publication 68.4% (*n* = 175/256) started pertuzumab. Due to the small number of women, it was not feasible to look further at variation in use. As pertuzumab was accessible via the CDF prior to NICE TAG publication a MLME model was fitted in patients diagnosed 2014–2019. Age and comorbidity were found to be independently associated with differences in use. There was also evidence of increasing use with increasing year of diagnosis, but no evidence of variation by GOR (Table A1).

##### Adjuvant use for newly-diagnosed HER2-positive EIBC or LABC

Among 11,016 women who had surgery within six months of diagnosis for HER2-positive EIBC or LABC, with no prior treatment with pertuzumab or trastuzumab, 2.4% had a record of adjuvant pertuzumab. Use was higher for LABC (7.0%; Table 2A). 93.5% also had a record of adjuvant chemotherapy and trastuzumab.

**Fig. 3:**
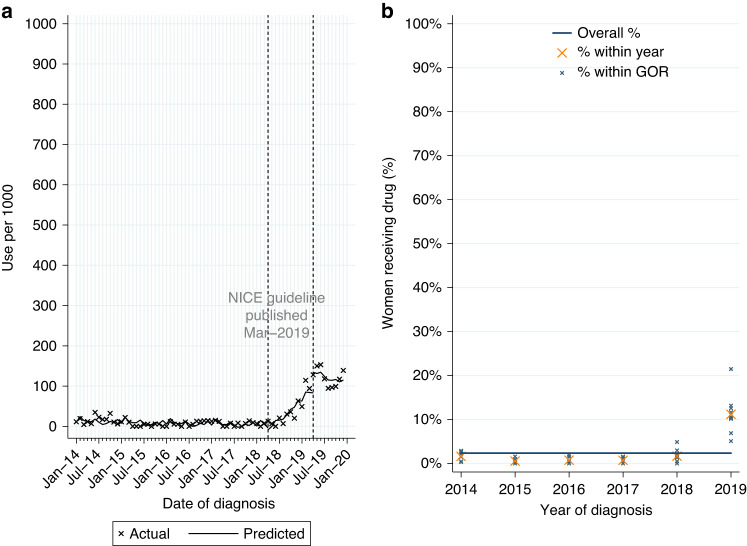
Adjuvant pertuzumab utilisation. Adjuvant pertuzumab* utilisation among women diagnosed with HER2-positive early invasive or locally advanced breast cancer from 2014 to 2019, and receiving surgery within 6 m of diagnosis. **a** Interrupted time series analysis of monthly adjuvant pertuzumab* initiations within 6 m of surgery. **b** Observed percentage of women initiating adjuvant pertuzumab*, by Government Office Region (GOR) and year of diagnosis. *with no prior trastuzumab or pertuzumab received before surgery.

From ITSA we found monthly initiations of adjuvant pertuzumab increased by 8.1 patients per 1000, among eligible women diagnosed between April 2018 and March 2019 (Table A3, *p* < 0.0001; Fig. 3a), with an immediate increase in use among women diagnosed post-publication (*p* = 0.017). This increase was observed across all GORs (Fig. 3b). There was no evidence of continued increase in use among women diagnosed from April 2019 onwards (*p* = 0.230).

Among eligible women diagnosed from April 2019 onwards 12.1% started adjuvant pertuzumab, with higher use for LABC (28.3%; Table 2A). Fitting a MLME model, age and GOR (observed range: 3.0–22.0%; Table A2) were both independently associated with variation in use (Table A1). Low use among women aged 80+ was observed across all GORs (Fig. A3b).

#### Ribociclib or palbociclib

Ribociclib and palbociclib are CDK4/6 inhibitors recommended by NICE in 2017 for HER2-negative, hormone receptor-positive LABC or MBC. Among eligible women diagnosed post-publication 2.2% (*n* = 44/1962) had a record of ribociclib; 81.8% of use was for MBC. Numbers were insufficient to further investigate uptake of ribociclib.

Among women newly-diagnosed with HER2-negative, hormone receptor-positive LABC or MBC from 2014 to 2019, 9.7% (*n* = 591/6104) had a record of palbociclib, increasing to 23.2% (*n* = 455/1962) among women diagnosed post-publication. Use was highest for MBC (Table 2A). Of those starting palbociclib 93.6% (*n* = 88/94) of women with LABC and 89.9% (*n* = 447/497) of women with MBC also had a record of ever being prescribed an aromatase inhibitor. Among women with MBC, over three-quarters started pablociclib within 4 months of diagnosis.

**Fig. 4:**
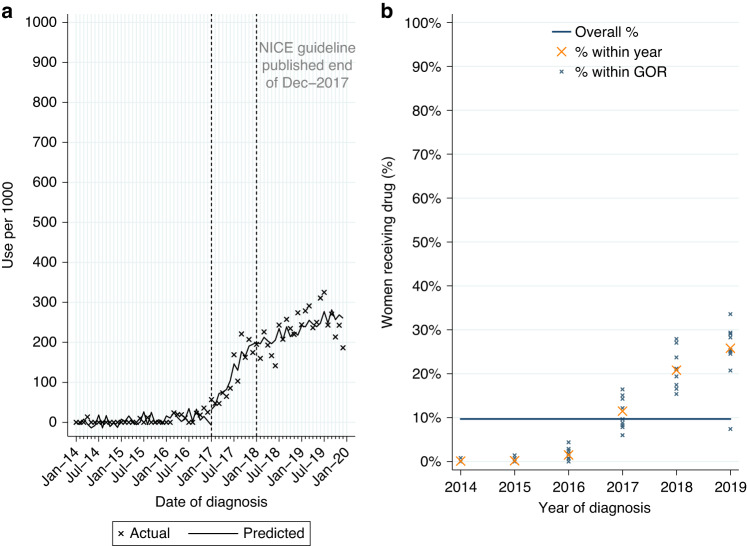
Palbociclib utilisation. Palbociclib utilisation among women initially diagnosed with HER2-negative, hormone receptor-positive metastatic breast cancer from 2014 to 2019. **a** Interrupted time series analysis of monthly palbociclib initiations. **b** Observed percentage of women initiating palbociclib, by Government Office Region (GOR) and year of diagnosis.

From ITSA we found use of palbociclib increased among eligible women diagnosed during the 12 months prior to NICE TAG publication in December 2017 (monthly increase of 16.7 per 1000 patients, Table A3, *p* < 0.0001; Fig. 4a). Use continued to increase among women diagnosed from January 2018 onwards (monthly increase of 3.5 per 1000 patients, *p* = 0.007).

Among eligible women diagnosed post-publication, use varied by stage at diagnosis (6.2% LABC; 43.8% MBC; Table 2A). Fitting a MLME model, age was found to be independently associated with variation in use, but not comorbidity or GOR (Table A1). Use had increased over time across GORs (Fig. 4b) and for all age groups (Fig. A4a) but was lower among women aged 80+ years in all GORs (Fig. A4b).

#### Abemaciclib

Abemaciclib was recommended for use in 2019, 14 months after publication of NICE TAGs for palbociclib and ribociclib. Use among eligible women diagnosed after February 2019 was 2.3% (*n* = 18/795), with highest use for MBC. Numbers were insufficient to investigate uptake of abemaciclib further.

### Uptake of drugs recommend by NICE for use following relapse/recurrence, progression or previous treatment

Table 2B describes the percentage of eligible women, among those who did not die within 12 months of diagnosis, with a record of starting each of the drugs recommended by NICE for use beyond a first-line setting or following initial treatment. For all drugs, post-publication use was highest among women aged 50–69 years and those with MBC (where use was recommended across stage groups). The findings for each drug are presented in the following sections.

#### Gemcitabine

Gemcitabine was recommended by NICE in January 2007 for MBC. 1.2% (*n* = 70/5739) of eligible women had a record of ever starting gemcitabine. There was no use among women aged 80+ years.

#### Everolimus or Eribulin

Everolimus (an mTOR kinase inhibitor for HER2-negative, hormone receptor-positive MBC) and eribulin (a chemotherapy for LABC or MBC) were both recommended by NICE in December 2016. Among eligible women diagnosed from 2017 to 2019, 4.4% (*n* = 55/1250) had a record of everolimus, and 2.0% (*n* = 122/6117) had a record of eribulin.

#### Trastuzumab-emtansine

Trastuzumab-emtansine, a HER2-targeting drug, was recommended for LABC or MBC in July 2017 for use in patients previously treated with trastuzumab and a taxane-based regimen. Among eligible women diagnosed August 2017–December 2019, 12.7% (*n* = 87/684) had a record of trastuzumab-emtansine. Use was highest for MBC (18.9%) compared with LABC (7.0%). Rates of use for MBC were highest in northern geographical regions (observed range across all regions: 5.6–33.3%).

#### Neratinib

Neratinib is another HER2-targeting therapy, recommended for HER2-positive, hormone receptor-positive EIBC by NICE in November 2019, for use following previous treatment. There were 114 eligible women diagnosed in December 2019, of whom 3.5% (*n* = 4) had a record of neratinib. There was no use among women aged 70+ years.

## Discussion

This population-based study used routinely-collected clinical data to evaluate the use of NICE-recommended drugs. Data were available for more than 160,000 women aged 50+ years diagnosed with IBC in England from 2014 to 2019.

Thirteen NICE TAGs were published between March 2002 and November 2019 where NICE made a positive recommendation. Several drug types, including HER2-targeting therapies, CDK 4/6 inhibitors and chemotherapies, were recommended for use either as part of first-line treatment or following recurrence/progression/previous treatment. Where use was recommended across stage groups, recorded use increased as stage group increased, with highest rates for MBC (where treatment was indicated). Use varied by age at diagnosis. Where numbers allowed for further analysis (trastuzumab, pertuzumab and palbociclib) there was evidence that differences by age were independent of other factors including comorbidity burden and geographical region. There are likely multiple reasons for this, including a lack of robust evidence for the efficacy and tolerability of treatments among older patients, who are under-represented in clinical trials. This might have led to a reluctance among oncologists to use new therapies for older patients. Other publications have reported reduced use of treatment in older patients irrespective of comorbidity [24, 25].

Where NICE-recommended drugs were intended for first-line treatment, utilisation among eligible women diagnosed post-publication varied, with 63.8% recorded as having HER2-targeting therapies (trastuzumab/pertuzumab), compared to 26.6% for CDK 4/6 inhibitors (ribociclib; palbociclib; abemaciclib). Highest rates of recorded drug use were for trastuzumab, a drug first introduced into clinical practice two decades ago and added to the World Health Organization Model List of Essential Medicines in 2015 [26]. A change in use to trastuzumab biosimilars for some patients may also have contributed to high use among women diagnosed in 2018 and 2019, demonstrating the continuing influence of drug development and approvals on uptake of existing approved drugs. This finding is echoed in an Italian study which reported increased use, with trastuzumab biosimilars contributing to around one-third of trastuzumab-based treatment among patients diagnosed in more recent years [27]. Additionally a review of biosimilars highlighted the value of their inclusion in trastuzumab-based treatment in increasing access to anti-HER2 therapies, particularly in relation to cost-saving [28].

Of three recommended CDK4/6 inhibitors, palbociclib was predominantly used, accounting for 87.2% of CDK4/6 inhibitor-based first-line treatment. This may be explained in part by palbociclib’s existing approvals by the American Food and Drug Administration (FDA) (2015) and European Medicines Agency (EMA) (2016), whereas FDA/EMA approvals for ribociclib were in the same year as the NICE approval. Use of neoadjuvant/adjuvant pertuzumab and palbociclib, drugs recommended for first-line use by NICE between 2014 and 2019, increased among women diagnosed in the months around NICE TAG publication. The increase among women diagnosed 6–12 months prior to publication will be in part due to the timing of treatment in relation to diagnosis but may also be due to these drugs being previously approved for use by the EMA. Typically drugs with a new therapeutic indication have been approved first by the FDA in the US, followed by the EMA for use in Europe [29, 30]. Although the EMA provides market authorisation for all drugs to be used across Europe, within the UK NHS setting it is only following publication of the NICE TAG that they are usually recommended for use in routine practice [1]. There was no evidence that there was a national-level change in use following NICE recommendation of pertuzumab for MBC, however this drug was already available to patients through the NHS Cancer Drugs Fund (CDF). The CDF is another means through which oncological drugs with insufficient evidence of benefit at the point of appraisal are made available to patients [31–33]. Access via the CDF and a subsequent NICE recommendation meant there was an increase in use over the study period.

Where drugs were recommended for use beyond a first-line setting or following previous treatment, rates of recorded use were generally low. Patterns of use among women diagnosed post-publication were similar to those observed for first-line treatment, with use decreasing as age increased and highest for MBC.

There have been few previous studies looking at the translation of nationally recommended oncological drugs for IBC into routine care, with studies focusing on the safety and effectiveness of new drugs [34–40]. A study in the US identified a marked increase in the use of oral cancer drugs with no documented overall survival benefit between 2011 and 2018 [41].

There are several strengths of this study. First, it provides real-world evidence of utilisation in routine care by using routinely-collected national data available for all women aged 50 years and over with a registered diagnosis of IBC in England from 2014 to 2019. Second, linked patient-level data on drug utilisation were available up to 28 February 2022, providing good follow-up (at least 26 months). Third, it provides robust estimates of drug utilisation as the study used drug information captured in SACT. SACT is a national dataset of systemic therapy in cancer, with whole population coverage in England and 100% data completeness for the data items used in this study (drug name and administration date) [11, 42].

We are aware there are also some limitations. First, it was not possible to provide estimates of the use of NICE-recommended drugs in women aged under 50 years who had IBC, as data were only available for women aged 50 years and over. Second, data on hormone receptor status, HER2 status and stage were typically less complete as age increased. As this information is provided to NCRAS through an automatic pathology feed it is likely that lower completeness is reflective of a lack of testing of molecular markers which therefore are not available to inform treatment decisions in this group of older women. This should therefore not impact our findings on the rates of treatment use among different age groups defined according to this information. Third, as SACT data returns may be low for some NHS trusts estimates for the use of new drugs for recently diagnosed patients may be lower than in reality. Finally, it was difficult to define cohorts of eligible patients within the routine data for drugs recommended for use following progression, relapse, recurrence or previous treatment. The study cohort, representing relatively recently diagnosed cases is less likely to provide a reliable estimate of the use of these drugs in these clinical settings, and may underestimate their use in the overall population. In addition to this there was low utilisation of some drugs meaning that analysis of variation in use was not possible. This is something which would benefit from further research in the future, to identify any barriers to access.

For ribociclib and abemaciclib, the study found insufficient uptake following the NICE TAG publication to carry out ITSA and provide robust estimates of the impact of NICE guidance on utilisation. Future research should evaluate longer-term uptake of NICE-recommended drugs and carry out ITSA to assess the impact of NICE TAG publication on use. Additionally future work to understand the extent of non-concordant use of NICE-recommended drugs would provide further insight into the drugs investigated within this study.

## Conclusions

The translation of evidence from trials into routine care, beyond recommendations made in national treatment guidelines, is difficult to study but is of profound importance in efforts to improve population health. The findings of this population-based study looking at uptake of oncological drugs highlight varied utilisation of treatments recommended by NICE for IBC within the last 20 years. Additionally it highlights lower use of NICE-recommended drugs for first-line treatment as age increased, regardless of geographical region or comorbidity burden. Future work should further investigate geographical variation in access to new drugs, to identify areas of the country where routine access to new drugs is below what would be expected. Improving access to effective treatments is an important step in understanding IBC outcomes. At organisation level, NHS trusts are encouraged to perform local audit of NICE-recommended drugs to ensure patient fitness for treatment is assessed and age is not a barrier to access. Providing patients with clear information on NICE-recommended drugs may also improve engagement in decision-making where this is a contributing factor.

### Supplementary information

Gannon_BJC2023_NICE_Drugs_Breast_Cancer Supplementary Material

Gannon_BJC2023_NICE_Drugs_Breast_Cancer - STROBE_checklist_cohort

## Data Availability

This work uses data that has been provided by patients and collected by the NHS as part of their care and support. The data for England are collated, maintained and quality assured by the National Disease Registration Service (NDRS), which is part of NHS England. Data on English Cancer Registrations can be accessed via the NHS Digital Data Access request Service (DARS) https://digital.nhs.uk/services/data-access-request-service-dars#national-disease-registration-service-ndrs.

## Code availability

All analyses were carried out using standard methods therefore the code is not provided.
