# Supplementary material for: Evidence into practice: a national cohort study of NICE-recommended oncological drug therapy utilisation among women diagnosed with invasive breast cancer in England
Source: Br J Cancer. 2023 Sep 23;129(10):1569–79. doi: 10.1038/s41416-023-02439-z (PMC10646146; doi:10.1038/s41416-023-02439-z)
Supplement: Supplementary file 1 — Gannon_BJC2023_NICE_Drugs_Breast_Cancer Supplementary Material [file 41416_2023_2439_MOESM1_ESM.pdf]

Gannon et al. BJC 2023.

# Evidence into practice: A national cohort study of NICE-recommended oncological drug therapy utilisation among women diagnosed with invasive breast cancer in England.

Supplementary Material

## Appendix 1:

Steps taken to identify published NICE Technology Appraisal Guidance (TAGs) within https://www.nice.org.uk/guidance . Performed on 17/03/2022.
Step 1: Select "Technology Appraisal Guidance".
Step 2: Ensure you are on the "Published" tab.
Step 3: Apply filters. Last updated date: From date = "01/01/2000"; To date = "31/12/2019". Type: select "Guidance". Guidance programme: select "Technology appraisal guidance". Filter by title or keyword: type "breast cancer".

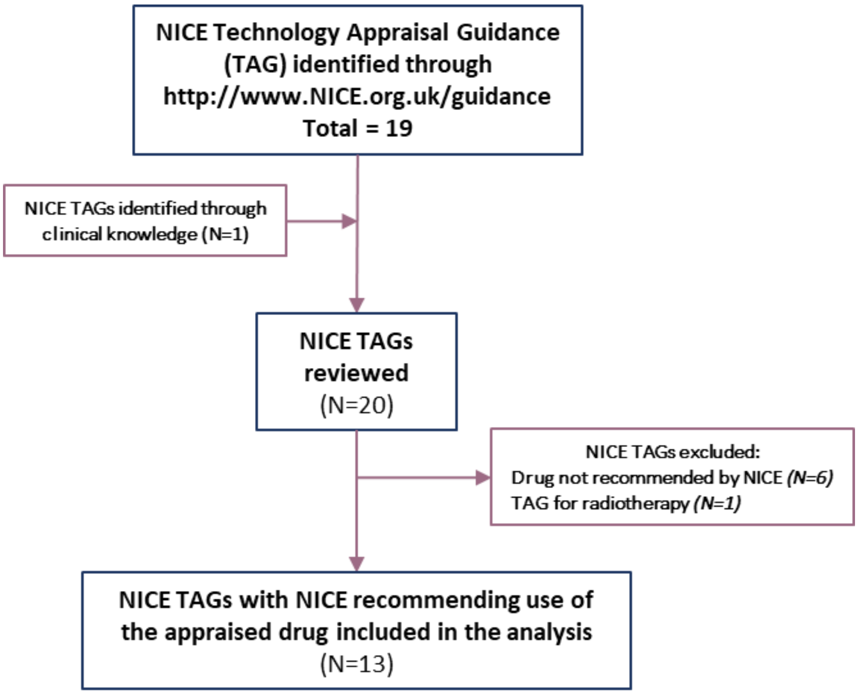


**NICE Technology Appraisal Guidance excluded from the study.**

| Reference | Reason for exclusion |
|---|---|
| NICE. Bevacizumab in combination with a taxane for the first-line treatment of metastatic breast cancer. 2011 [Available from: https://www.nice.org.uk/guidance/ta214 ] | Drug not recommended by NICE |
| NICE. Fulvestrant for the treatment of locally advanced or metastatic breast cancer. 2011 [Available from: https://www.nice.org.uk/guidance/ta239 ] | Drug not recommended by NICE |
| NICE. Lapatinib or trastuzumab in combination with an aromatase inhibitor for the first-line treatment of metastatic hormone-receptor-positive breast cancer that overexpresses HER2. 2012 [Available from: https://www.nice.org.uk/guidance/ta257 ] | Drug not recommended by NICE |
| NICE. Bevacizumab in combination with capecitabine for the first-line treatment of metastatic breast cancer. 2012 [Available from: https://www.nice.org.uk/guidance/ta263 ] | Drug not recommended by NICE |
| NICE. Fulvestrant for untreated locally advanced or metastatic oestrogen-receptor positive breast cancer. 2018 [Available from: https://www.nice.org.uk/guidance/ta503 ] | Drug not recommended by NICE |
| NICE. Intrabeam radiotherapy system for adjuvant treatment of early breast cancer. 2018 [Available from: https://www.nice.org.uk/guidance/ta501 ] | TAG for radiotherapy not an oncological drug |
| NICE. Eribulin for treating locally advanced or metastatic breast cancer after 1 chemotherapy regimen. 2018 [Available from: https://www.nice.org.uk/guidance/ta515 ] | Drug not recommended by NICE |

## Appendix 2:

**Figure A1:** Details of patient selection from women aged 50 and over, diagnosed with breast cancer in a NHS trust in England, between 2014 and 2019.

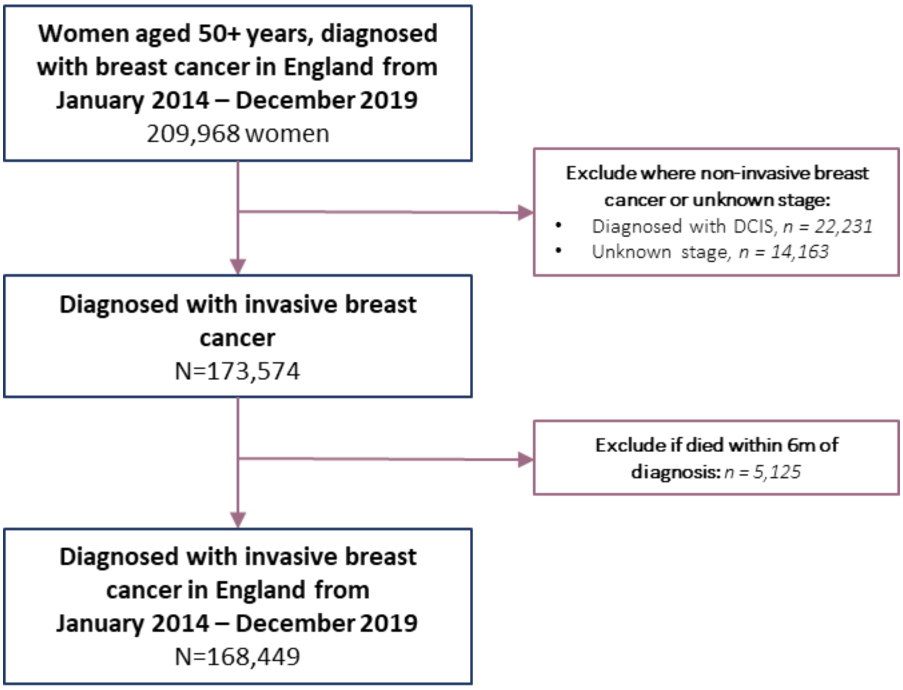

**Figure A2.** Trastuzumab utilisation among women initially diagnosed with HER2-positive early invasive breast cancer (EIBC), locally advanced breast cancer (LABC) or metastatic breast cancer (MBC) from 2014–2019

**Figure A2a.** Line plot of trastuzumab initiations, per 1000 women, by date of diagnosis

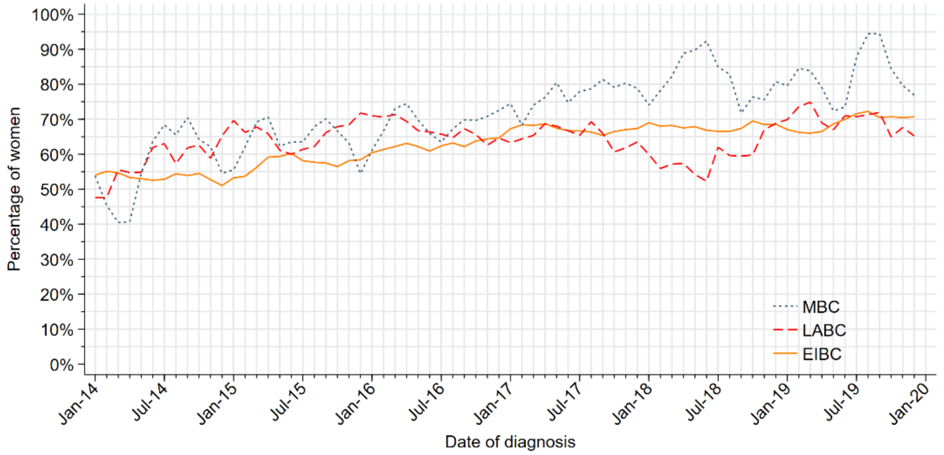


***Note**: line plot created using tsline, with smoothed moving average using uniform weights. Trastuzumab initiation includes trastuzumab (Herceptin) and trastuzumab biosimilars.*

**Figure A2b.** Line plot of trastuzumab biosimilar initiations, per 1000 women, by date of diagnosis

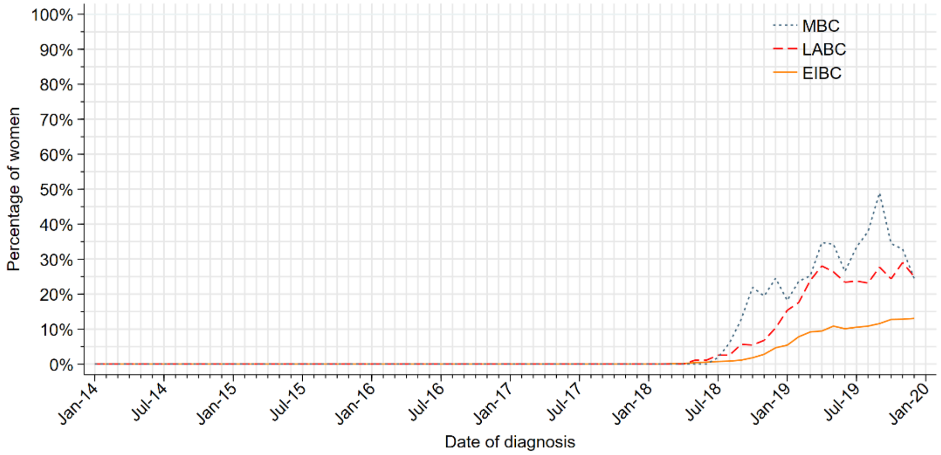


***Note**: line plot created using tsline, with smoothed moving average using uniform weights.*

**Figure A2c.** Observed percentage of women initiating trastuzumab, by Government office Region (GOR) and age at diagnosis

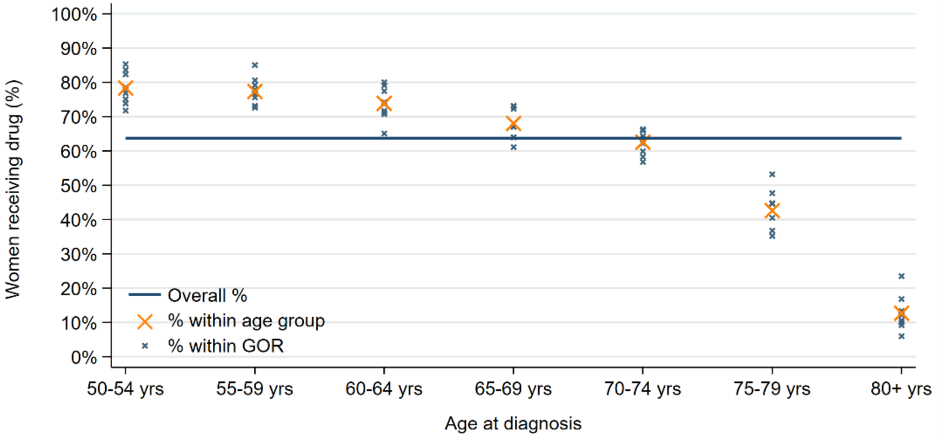


**Figure A2d.** Observed percentage of women initiating trastuzumab, by Government office Region (GOR) and age at diagnosis

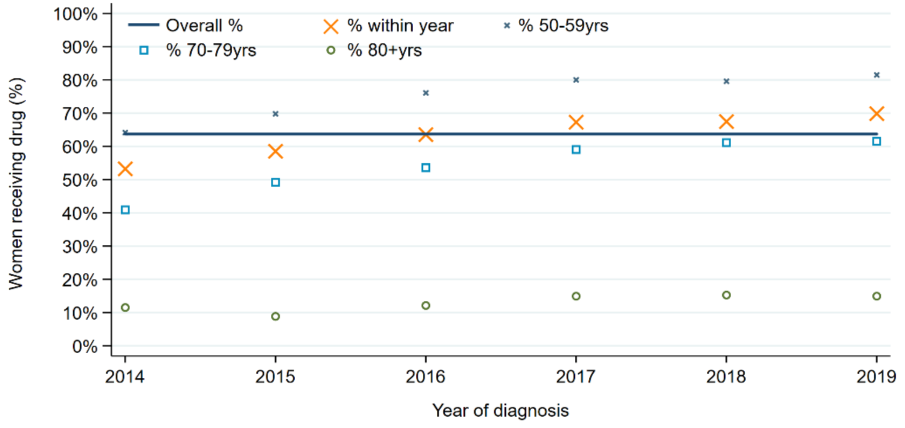

**Figure A3.** Post-publication pertuzumab utilisation among women initially diagnosed with HER2-positive early invasive breast cancer (EIBC) or locally advanced breast cancer (LABC)

**Figure A3a.** Observed percentage of women initiating neoadjuvant pertuzumab for EIBC or LABC, by Government office Region (GOR) and age at diagnosis

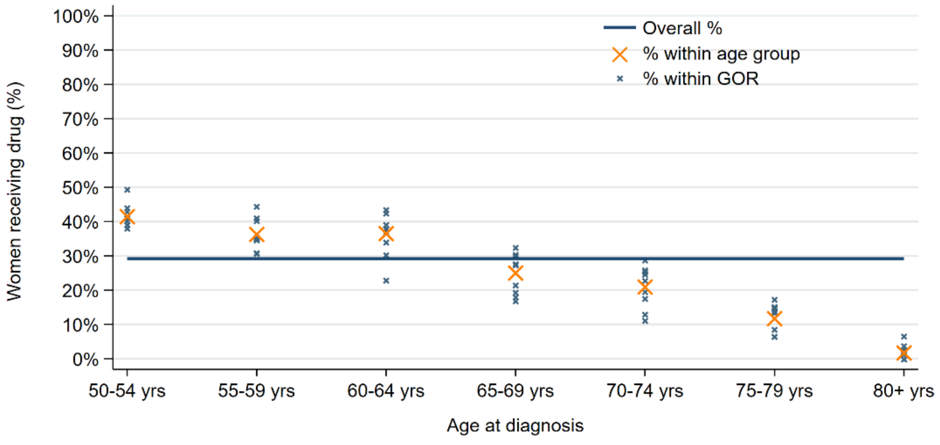


**Figure A3b.** Observed percentage of women initiating adjuvant pertuzumab for EIBC or LABC, by Government office Region (GOR) and age at diagnosis

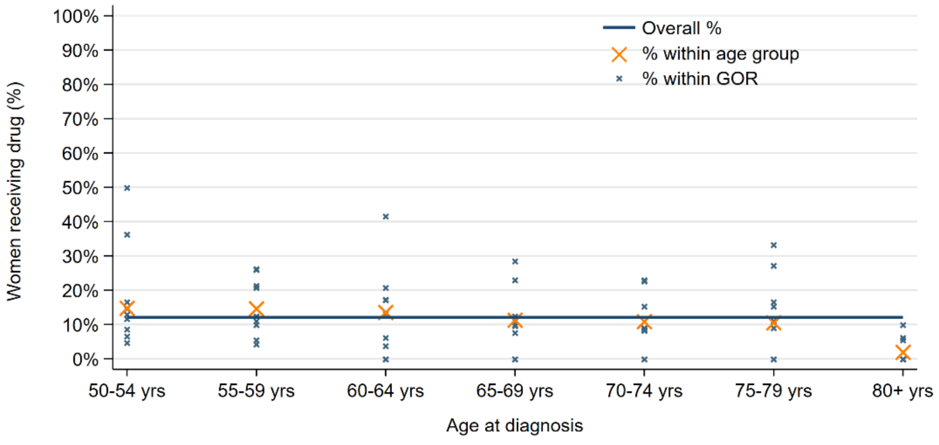


**Figure A4.** Post-publication palbociclib utilisation among women initially diagnosed with HER2-positive locally advanced breast cancer (LABC) or metastatic breast cancer (MBC)

**Figure A4a.** Observed percentage of women initiating palbociclib for LABC or MBC, by year of diagnosis and age at diagnosis

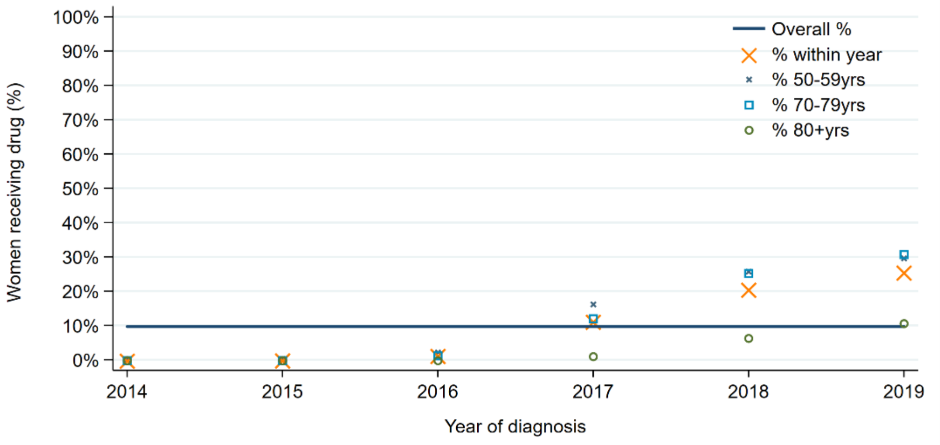


**Figure A4b.** Observed post-publication percentage of women initiating palbociclib for LABC or MBC, by Government office Region (GOR) and age at diagnosis

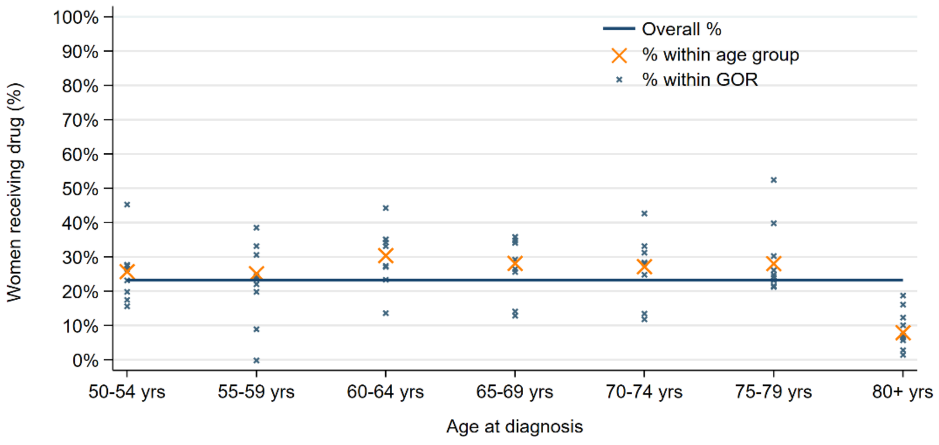

**Table A1:** Adjusted odds ratios of drug use recorded within SACT, for oncological treatments for breast cancer recommended by NICE for use as first-line treatment following initial diagnosis, among eligible women

| Drug (NICE TAG date) | Indication/ Eligibility | Cohort of women diagnosed | Use % (n/N) | Adjusted* odds ratio (95% confidence interval) | | | | | | | | | |
|---|---|---|---|---|---|---|---|---|---|---|---|---|---|
| | | | | 50-59 years | 60-69 years | 70-79 years | 80+ years | Overall p-value | CCI = 0 | CCI = 1 | CCI = 2+ | Overall p-value | GOR p-value |
| **Trastuzumab** | HER2+ EIBC/LABC/MBC | Jan2014-Dec2019 | 63.7% (10776/16987) | 1.00 | 0.73 (0.67-0.80) | 0.28 (0.25-0.03) | 0.03 (0.03-0.04) | <0.0001 | 1.00 | 0.64 (0.57-0.73) | 0.30 (0.25-0.37) | <0.0001 | <0.0001 |
| **Neoadjuvant Pertuzumab** | HER2+ EIBC/LABC | Jan2017-Dec2019 | 29.2% (2068/7083) | 1.00 | 0.78 (0.68-0.89) | 0.25 (0.21-0.30) | 0.02 (0.01-0.03) | <0.0001 | 1.00 | 0.55 (0.43-0.71) | 0.33 (0.21-0.51) | <0.0001 | <0.0001 |
| **Pertuzumab** | HER2+ MBC | Jan2014-Dec2019 | 52.0% (485/932) | 1.00 | 0.84 (0.56-1.26) | 0.23 (0.15-0.35) | 0.04 (0.02-0.08) | <0.0001 | 1.00 | 0.87 (0.52-1.44) | 0.46 (0.22-0.95) | 0.0025 | 0.1420 |
| **Adjuvant Pertuzumab** | HER2+ EIBC/LABC | Apr2019-Dec2019 | 12.1% (129/1068) | 1.00 | 1.23 (0.57-2.66) | 0.23 (0.11-0.47) | 0.03 (0.01-0.10) | <0.0001 | 1.00 | 0.53 (0.21-1.36) | 0.28 (0.08-1.01) | 0.0805 | 0.0102 |
| **Palbociclib** | HER2-, HR+ LABC/MBC | Jan2018-Dec2019 | 23.2% (455/1962) | 1.00 | 1.11 (0.79-1.57) | 0.97 (0.70-1.36) | 0.21 (0.13-0.32) | <0.0001 | 1.00 | 0.74 (0.52-1.07) | 0.64 (0.38-1.07) | 0.1245 | 0.1999 |

Key: *HER2 = human epidermal growth receptor 2; MBC = metastatic breast cancer; EIBC = early invasive breast cancer; LABC = locally advanced breast cancer; HR+ = hormone receptor-positive.*
**Odds ratio from multilevel mixed-effects logistic regression model, adjusting for age, comorbidity burden, diagnosis year, deprivation, ethnicity, stage group, tumour characteristics, geographical region.*

**Table A2.** Observed percentage of drug use recorded within SACT, for oncological treatments for breast cancer recommended by NICE for use as first-line treatment following initial diagnosis, among eligible women, by Government Office Region

| Government Office Region | Trastuzumab | | Neoadjuvant Pertuzumab | | | | Pertuzumab for MBC | | | | Adjuvant Pertuzumab | | | | Palbociclib | | | |
|---|---|---|---|---|---|---|---|---|---|---|---|---|---|---|---|---|---|---|
| | All use | | All use | | Post-publication use | | All use | | Post-publication use | | All use | | Post-publication use | | All use | | Post-publication use | |
| | N | % | N | % | N | % | N | % | N | % | N | % | N | % | N | % | N | % |
| North East | 1,232 | 58.4% | 1,076 | 11.3% | 439 | 24.6% | 62 | 38.7% | 14 | 64.3% | 888 | 1.5% | 68 | 11.8% | 464 | 11.2% | 157 | 26.8% |
| North West | 2,186 | 69.6% | 1,940 | 18.6% | 962 | 35.1% | 100 | 57.0% | 34 | 52.9% | 1,409 | 1.6% | 134 | 3.0% | 877 | 10.9% | 294 | 24.5% |
| Yorkshire & the Humber | 1,830 | 69.5% | 1,592 | 13.0% | 765 | 25.2% | 110 | 46.4% | 28 | 71.4% | 1,253 | 3.7% | 132 | 22.0% | 633 | 9.5% | 203 | 24.1% |
| East Midlands | 1,364 | 62.8% | 1,198 | 14.1% | 577 | 27.4% | 68 | 58.8% | 25 | 72.0% | 951 | 2.4% | 90 | 12.2% | 386 | 6.0% | 121 | 14.9% |
| West Midlands | 1,817 | 60.9% | 1,604 | 15.7% | 726 | 32.2% | 91 | 44.0% | 24 | 66.7% | 1,210 | 2.3% | 98 | 13.3% | 621 | 9.2% | 196 | 24.5% |
| East England | 2,168 | 62.3% | 1,846 | 16.4% | 910 | 30.9% | 139 | 47.5% | 35 | 65.7% | 1,335 | 1.3% | 116 | 8.6% | 800 | 8.8% | 247 | 21.9% |
| London | 1,783 | 60.4% | 1,478 | 16.1% | 751 | 27.4% | 109 | 60.6% | 23 | 78.3% | 1,054 | 2.8% | 116 | 12.9% | 661 | 10.0% | 186 | 22.0% |
| South East | 2,607 | 64.5% | 2,247 | 14.9% | 1,111 | 26.5% | 143 | 53.1% | 41 | 68.3% | 1,700 | 2.6% | 192 | 11.5% | 944 | 10.8% | 323 | 23.8% |
| South West | 1,910 | 61.9% | 1,652 | 16.8% | 842 | 30.4% | 110 | 59.1% | 32 | 78.1% | 1,216 | 2.8% | 122 | 13.9% | 718 | 9.1% | 235 | 23.0% |

**Table A3.** Full interrupted time series analysis output of drug initiations (per 1000)

| Time period | Drug *(associated figure)* | Coefficient | 95% confidence interval | | P-value |
|---|---|---|---|---|---|
| | **Neoadjuvant pertuzumab** *(Figure 1a)* | | | | |
| Ref | Drug initiations, per 1000, in Jan-2014 | 2.8 | -17.1 | 22.7 | 0.779 |
| Ref | Monthly change in drug initiations, per 1000, from Jan-2014 to Jun-2016 | 0.4 | -0.1 | 0.9 | 0.087 |
| T | Immediate change in drug initiations, per 1000, in Jul-2016 (6m prior to NICE TAG publication) | 5.9 | -20.4 | 32.1 | 0.657 |
| T | Monthly change in drug initiations, per 1000, from Jul-2016 to Dec-2016 | 37.2 | 19.6 | 54.9 | <0.0001 |
| PP | Immediate change in drug initiations, per 1000, in Jan-2017 (month following NICE TAG publication in Dec-2016) | 15.9 | -80.9 | 112.7 | 0.743 |
| PP | Monthly change in drug initiations, per 1000, from Jan-2017 onwards | 1.9 | 0.9 | 2.9 | <0.0001 |
| | | | | | |
| | **Pertuzumab for MBC** *(Figure 2a)* | | | | |
| Ref | Drug initiations, per 1000, in Jan-2014 | 292.2 | 224.0 | 360.3 | <0.0001 |
| Ref | Monthly change in drug initiations, per 1000, from Jan-2014 to Mar-2017 | 6.9 | 4.0 | 9.7 | <0.0001 |
| T | Immediate change in drug initiations, per 1000, in Apr-2017 (12m prior to NICE TAG publication) | 92.5 | -80.4 | 265.3 | 0.288 |
| T | Monthly change in drug initiations, per 1000, from Apr-2017 to Mar-2018 | -1.1 | -22.9 | 19.9 | 0.915 |
| PP | Immediate change in drug initiations, per 1000, in Apr-2018 (month following NICE TAG publication in Mar-2018) | 23.9 | -181.4 | 229.1 | 0.817 |
| PP | Monthly change in drug initiations, per 1000, from Apr-2018 onwards | 5.8 | -6.1 | 17.7 | 0.332 |
| | | | | | |
| | **Adjuvant pertuzumab** *(Figure 3a)* | | | | |
| Ref | Drug initiations, per 1000, in Jan-2014 | 10.4 | 1.0 | 19.8 | 0.030 |
| Ref | Monthly change in drug initiations, per 1000, from Jan-2014 to Mar-2018 | -0.2 | -0.4 | 0.0 | 0.060 |
| T | Immediate change in drug initiations, per 1000, in Apr-2018 (12m prior to NICE TAG publication) | -10.5 | -30.1 | 9.1 | 0.289 |
| T | Monthly change in drug initiations, per 1000, from Apr-2018 to Mar-2019 | 8.1 | 4.7 | 11.6 | <0.0001 |
| PP | Immediate change in drug initiations, per 1000, in Apr-2019 (month following NICE TAG publication in Mar-2019) | 45.1 | 8.5 | 81.8 | 0.017 |
| PP | Monthly change in drug initiations, per 1000, from Apr-2019 onwards | -3.3 | -8.7 | 2.1 | 0.230 |
| | | | | | |
| | **Palbociclib** *(Figure 4a)* | | | | |
| Ref | Drug initiations, per 1000, in Jan-2014 | -0.2 | -13.9 | 13.5 | 0.979 |
| Ref | Monthly change in drug initiations, per 1000, from Jan-2014 to Dec-2016 | 0.7 | 0.1 | 1.3 | 0.024 |
| T | Immediate change in drug initiations, per 1000, in Jan-2017 (12m prior to NICE TAG publication) | 7.8 | -23.1 | 38.7 | 0.615 |
| T | Monthly change in drug initiations, per 1000, from Jan-2017 to Dec-2017 | 16.7 | 12.2 | 21.1 | <0.0001 |
| PP | Immediate change in drug initiations, per 1000, in Jan-2018 (month following NICE TAG publication in Dec-2016) | -32.9 | -79.0 | 13.2 | 0.158 |
| PP | Monthly change in drug initiations, per 1000, from Jan-2018 onwards | 3.5 | 1.0 | 6.1 | 0.007 |

Key: Ref = pre-publication time period, T = Transition period, PP = post-publication period

The coefficients in the reference (pre-publication) period are the baseline rate and monthly trend. The coefficients for the transition and post-publication periods are the change in the rate in the first month of the period and the trend for that period.
